# Do cardiovascular disease prevention programs in northern Sweden impact on population health? An interrupted time series analysis

**DOI:** 10.1186/s12889-019-6514-x

**Published:** 2019-02-15

**Authors:** Miguel San Sebastián, Paola A. Mosquera, Per E. Gustafsson

**Affiliations:** Department of Epidemiology and Global Health, Umeå University, SE-901 87 Umeå, Sweden

**Keywords:** Ischemic heart disease, Morbidity, Mortality, Interrupted time series analysis, Intervention, Northern Sweden

## Abstract

**Background:**

Cardiovascular disease (CVD) is the main cause of morbidity and mortality in Sweden. This study aims to assess the impact of a CVD intervention implemented in 1993 in northern Sweden on the reduction of premature ischemic heart disease (IHD) morbidity and mortality in women and men during the period 1987–2013.

**Methods:**

An ecological controlled interrupted time series design, with pre-intervention period defined as 1987–1993 and post-intervention period 1994–2013 was carried out. For each year, IHD events, stratified by sex, were retrieved from national registers.

**Results:**

Impressive reductions on IHD premature morbidity and mortality were observed to a similar degree in both the intervention county and the other comparison counties across the last 27 years. Significant differences in the pre-post intervention trends indicating the intervention group had smaller reductions than expected from its pre-intervention trend and the trend of control counties were found among men for both IHD morbidity and mortality. A similar pattern was observed among women but without significant differences.

**Conclusions:**

Taken together, the data do not support that the intervention has contributed to an additional reduction on IHD morbidity and mortality, above and beyond that which is already seen in neighbouring counties without similar programs.

**Electronic supplementary material:**

The online version of this article (10.1186/s12889-019-6514-x) contains supplementary material, which is available to authorized users.

## Background

Although cardiovascular disease (CVD) continue to be the main cause of morbidity and mortality in Sweden, important reductions have been observed in the last two decades [1, 2]. As an illustration, coronary heart disease mortality rates decreased from 1987 to 2009 by 67.4% in men and 65.1% in women aged 35 to 84 [1]. This promising development is likely explained by the combination of an improved risk factor profile in the population, such as decreases in serum cholesterol, smoking rates and blood pressure, and improvements in medical interventions including better treatment of acute myocardial infarction and secondary prevention [1, 3].

Measures to reduce the burden of CVD have been of high priority in Sweden. Numerous programs and initiatives have been implemented by the Swedish National Institute of Public Health and many are currently ongoing [4], representing a mixture of both community- and individual-based strategies.

Community-based CVD prevention programs are based on the idea that if the population as a whole is targeted, even a modest risk factor and lifestyle change would potentially have a substantial public health impact [5]. Such programs have a long tradition in high income countries, and have been implemented to promote risk-reducing lifestyle changes in different populations since the 1970s [6, 7]. However, a review has highlighted that whereas community programs for prevention of CVDs seem to achieve favorable changes in overall CVD risk factors, this may not necessarily be reflected in decreased mortality [8].

Individual-based strategies, in contrast, commonly take the form of periodical health examinations (also known as health checks), which involve systematic screening of non-symptomatic adult population and behavioural counselling and/or treatment to those at high risk [9]. Albeit an established public health strategy, the actual contribution of such health checks to CVD risk reduction and the appropriateness of approaches based on identifying high-risk individuals, are not exempt of controversy [10–12]. For example, a recent Cochrane systematic review found no evidence that general health checks reduced morbidity or mortality, and the review therefore concluded that this type of practices should be avoided [13].

In Sweden, the northernmost counties (collectively referred to as Norrland) have traditionally shared the highest CVD mortality rates in the country. As a response, the Västerbotten county council initiated in the mid-1980s an intervention program aiming to reduce CVD and diabetes morbidity and mortality. This program, the Västerbotten Intervention Program (VIP), was piloted in a small municipality in 1985 and gradually expanded over the years to finally cover all municipalities in Västerbotten in 1993 [14]. The VIP combined a population-based strategy directed towards the general public with an individual-based strategy aimed at reaching all middle-aged persons by inviting them to participate in screening and health counselling conducted by their primary health care providers. Examples given in the literature of the community-based activities include meetings with the population with discussions about public health issues, information in local media, in schools and at workplaces about health promotion and healthy lifestyles, NGO activities and labelling of healthy foods in local stores [14]. While the community component was the responsibility of the municipalities and has followed varying intensities and activities, the individual approach has followed a detailed systematic procedure over the course of the program [14].

Since the implementation of VIP, different trends in risk factors for CVDs have been observed among its participants, including increased Body Mass Index (BMI) and glucose, initial decreases in the prevalence of hypercholesterolemia but increasing trends during 2008–2010, and increased physical activity [15–18]. A recent study also suggested a reduction in all-cause and CVD mortality in the VIP participants and the eligible county population when compared to a national reference population [19]. However, no study has so far evaluated whether the VIP actually has resulted in improved population health in Västerbotten as a whole, an evaluation parameter that should be considered key for a program designed to reduce CVD morbidity and mortality in the population. Given the complexity of this kind of interventions and the debate about their effectiveness, more evaluative approaches are therefore needed to understand its potential impact, particularly at the population level.

With this in mind, our study assessed the impact of the VIP, fully implemented in 1993, on the reduction of premature ischemic heart disease (IHD) morbidity and mortality in women and men in the entire Västerbotten county population aged 40–74 years during the period 1987–2013, as compared to neighboring counties by using an interrupted time series analysis.

## Methods

### Setting

Northern Sweden occupies 60% of the Swedish land area but includes only 12% of its population, and 80% of the 44 municipalities are rural. The region has a historical dependence on mining, steel and forestry industries, and a nationally high prevalence of e.g. cardiovascular disease [20]. Four counties comprise this northern region: Norrbotten, Västerbotten, Västernorrland and Jämtland.

Starting as a local initiative in one municipality of Västerbotten 1985, the VIP was scaled up until covering all municipalities of Västerbotten in 1993. In the individual approach, all subjects living in Västerbotten county who become 40, 50 or 60 years are invited by their health care centers to participate in the VIP each year. Participants are requested to complete a comprehensive questionnaire covering socioeconomic and psychosocial conditions, health, and health behaviors. In addition, height and weight are measured and biomarkers such as glucose and cholesterol collected from blood samples. Each health survey concludes with a second visit to the health center, where the results of the examination and the questionnaire responses are discussed individually with a trained nurse. The dialogue, based on the method of motivational interviewing, covers all the participant’s risk factors and lifestyle habits with a focus on lifestyle modification depending on the risk factor identified. When appropriate, follow-up visits are recommended, and, referrals to the participant’s family doctor for further assessment and pharmacological treatment. Participation rates have remained relatively stable at 66–67% since 2005 [14].

Although other short-lived CVD intervention programs have been initiated e.g. in the 1980s, no intervention comparable to VIP with respect to longevity and population coverage has been implemented in the other three counties during the study period.

### Design and data source

The study utilized an ecological controlled interrupted time series (ITS) design, with pre-intervention period defined as 1987–1993 and post-intervention period 1994–2013. ITS is considered to be the strongest quasi-experimental research design, particularly suited to evaluate public health interventions introduced at a population level over a clearly defined time [21–23]. One of the main threats to validity in controlled interrupted time series analysis relates to time-varying confounding, such as changes in outcome coding, co-interventions, or changes in the population under study [21]. The use of a comparison group not exposed to the intervention helps to alleviate the validity concerns, therefore, the intervention county (Västerbotten) was compared to the neighboring counties (Norrbotten, Västernorrland and Jämtland counties).

For each year 1987–2013, IHD events, stratified by sex, were retrieved from the National Board of Health and Welfare’s publicly available IHD database based on the National Patient Register and Cause of Death Register (http://www.socialstyrelsen.se/statistik/statistikdatabas/hjartinfarkter). IHD events were defined as incident cases or deaths with acute heart attack or other ischemic disease as underlying cause for the population aged 40–74 years (ICD 9 = 410–414 until 1997; ICD 10 = I20-I25 since 1998), per year and 100,000 inhabitants. Population numbers in the 40–74 years group for each of the counties and at national level were obtained from the publicly available data in Statistics Sweden.

### Data analysis

Given the substantial sex/gender differences in prevalence and burden of different CVDs as well as in its risk factors [24–26], all the analyses were conducted separately for men and women. We first conducted a descriptive analysis calculating the average decrease in IHD morbidity and mortality (relative change in cumulative incidence (CI) from time 0 (t-1) to time 1 (t) expressed as a percentage; ΔCI = CI_t_ - CI_t-1_ / CI_t-1_ × 100) from the pre-intervention (1987–1993) to the post-intervention (1994–2013) period, for women and men in the intervention and control counties.

Subsequently, to compare the IHD trends in the intervention and control counties we used segmented regression analysis. When one or more control groups are available for comparison, as in the present design, a regression model including seven terms can be fitted as shown in the following equation [27]:

$$ {\mathrm{Y}}_{\mathrm{t}}={\upbeta}_0+{\upbeta}_1{\mathrm{T}}_{\mathrm{t}}+{\upbeta}_2{\mathrm{X}}_{\mathrm{t}}+{\upbeta}_3{\mathrm{X}}_{\mathrm{t}}{\mathrm{T}}_{\mathrm{t}}+{\upbeta}_4\mathrm{Z}+{\upbeta}_5{\mathrm{ZT}}_{\mathrm{t}}+{\upbeta}_6{\mathrm{ZX}}_{\mathrm{t}}+{\upbeta}_7{\mathrm{ZX}}_{\mathrm{t}}{\mathrm{T}}_{\mathrm{t}}+{\upvarepsilon}_{\mathrm{t}}. $$

where Y_t_ is the aggregated outcome variable measured at each equally-spaced time-point t, T_t_ is the time since the start of the study, and X_t_ is a dummy variable representing the intervention (pre-intervention period 0, post-intervention period 1). Z is a dummy variable indicating the cohort assignment (treatment or control; in this case Västerbotten vs the other Norrland counties), and ZT_t_, ZX_t_ and ZX_t_T_t_ are all interaction terms among the previously described variables.

Differences between the intervention and control group before, and therefore not attributable to the intervention are captured by β_4_ and β_5_. Specifically, β_4_ represents the intercept difference at the beginning of the study of the outcome variable between treatment and controls (initial mean level difference), and β_5_ the difference in the slope of the outcome variable between treatment and controls prior to the intervention (pre-trend difference). β_6_ indicates the mean level difference between treatment and control groups for the year immediately following the introduction of the intervention. Lastly, β_7_ estimates the impact of the intervention; the difference between treatment and control groups in the slope of the outcome after initiation of the intervention, compared to the pre-intervention (pre-post trend difference).

Additional estimates calculated and reported include the slope differences between treatment and control in the period following the initiation of the intervention (post-trend differences), i.e. equivalent to β_5_ when X_t_ = 1; and the pre-trend and post-trend slopes separately for the intervention and the control group.

Four models were built estimating the changes in level and trend in premature IHD morbidity and mortality, before and after the VIP intervention was fully implemented in 1993, separately by sex. Since the outcome was a count, a Poisson regression model was first used including the age population 40–74 years as the denominator to convert the outcome into a rate and adjust for any potential changes in the population over time. Initial analysis suggested a moderate degree of overdispersion, so a more flexible negative binomial model was used for all analyses. Robust standard errors for the parameters to control for mild violations of underlying assumptions were calculated. Rate ratios (RR) and the 95% confidence intervals (95% CI) were obtained using the Stata 13.0 software. Since the intervention was compared to a control group, differences below one indicate higher decreases in the intervention group compared to its counterpart.

Given that the impact on population’s health can take some time to occur and results can be sensitive to the choice of control group, sensitivity analyses were carried out using a) 1999 and 2004 instead of 1994 as cut-off time points for assessing the trends in all comparison groups; b) the two northern neighbouring counties as separate controls; and c) using national rates as controls; comprising eleven alternative analyses run per outcome and sex, in total 44 analyses. Results are presented in Additional file 1 for morbidity and mortality.

## Results

### IHD morbidity and mortality rates reduction

**Fig. 1:**
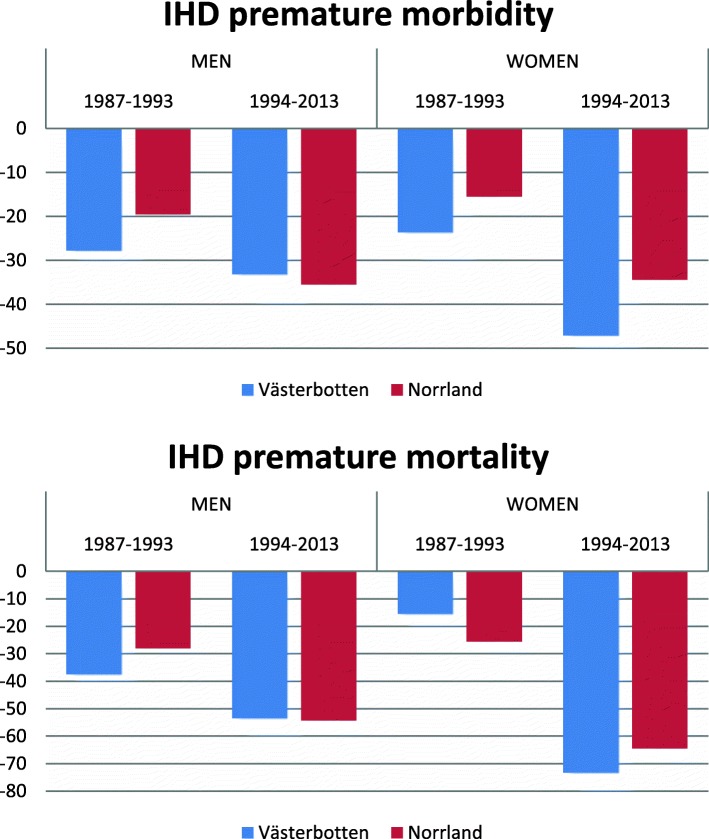
Percentage reduction of IHD morbidity and mortality by sex in Västerbotten (intervention county) and three control counties (Norrbotten, Västernorrland and Jämtland) during the periods of 1987–2013 and 1994–2013

A considerable reduction of IHD morbidity across the study period was observed in both the intervention (Västerbotten) and control (Norrbotten, Västernorrland and Jämtland) counties. During the pre-intervention period from 1987 to 1993, a numerically larger decrease in morbidity was observed in the intervention county compared to the control counties in both men (− 27.8% vs − 19.5%) and women (− 23.6% vs − 15.9%). In the post-intervention period from 1994 to 2013, the morbidity reduction was larger in the intervention county for women (− 47.1% vs − 34.4%) but not for men (− 33.2% vs − 35.5%) (Fig. 1).

Compared to the reductions in morbidity, an even greater relative decrease was seen for IHD mortality during the 27-year study period, In the pre-intervention period from 1987 to 1993, a higher mortality reduction was observed in the intervention than in the control counties among men (− 37.5% vs − 28.0%) but not among women (− 15.5% vs − 25.6%). In the post-intervention period from 1994 to 2013, the mortality reduction was similar the intervention and control counties among men (− 53.5% vs − 54.3), but with greater reduction in the intervention county among women (− 73.4% vs − 64.5%) (Fig. 1).

### Intervention impact on IHD morbidity

**Fig. 2:**
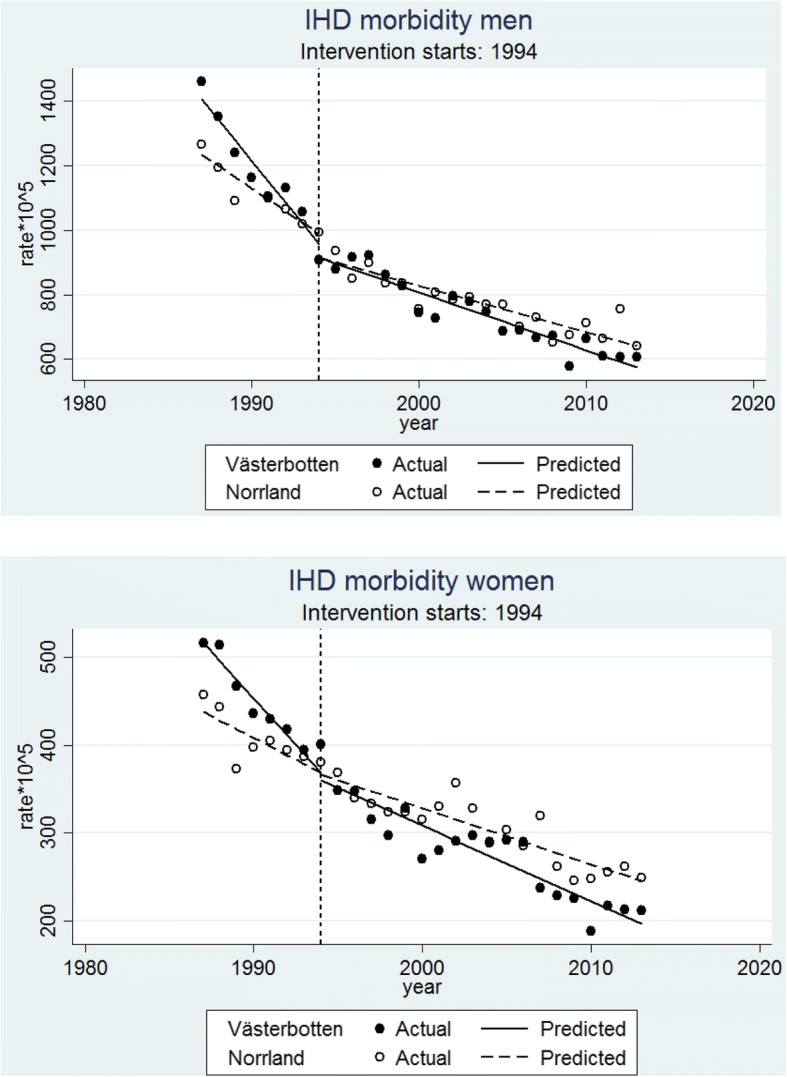
Premature Ischemic Heart Disease (IHD) morbidity (per 100,000 population) trends with fitted regression line before (1987–1993) and after (1994–2013) intervention started in the intervention county (Västerbotten) and control counties (Norrland; including Norrbotten, Västernorrland and Jämtland) in men (**a**) and women (**b**)

The results concerning morbidity are presented in Table 1 and Fig. 2. In men, at the beginning of the period (1987), Västerbotten started off with a numerically higher incidence than the three comparison counties of Norrland, as indicated by a significantly higher initial mean level (RR = 1.168; 95% CI = 1.095, 1.246). In the pre-intervention period, Västerbotten also displayed a 2.1% steeper decrease in morbidity compared to the control counties (pre-trend difference: RR = 0.979; 95% CI = 0.966, 0.992) (Table 1, Fig. 2). The steeper decline in morbidity in Västerbotten than control counties continued in the post-intervention period but of a substantially smaller magnitude of mere 0.6% difference (post-trend difference: RR = 0.994; 95% CI = 0.989, 0.999) (Table 1). This was also reflected in a significant pre-post-trend difference (RR = 1.016; 95% CI = 1.001, 1.030) reflecting that Västerbotten displayed a smaller post-intervention reduction in morbidity than would have been expected from its pre-intervention trend and the trends in the control counties.

**Table 1:** Segmented negative binomial regression analysis of IHD morbidity and mortality trends by sex comparing the county of Västerbotten (intervention) with three control counties (Norrbotten, Västernorrland and Jämtland) of Northern Sweden during the period 1987–2013

	IHD morbidity		IHD mortality	
	Men	Women	Men	Women
	RR (95% CI)	RR (95% CI)	RR (95% CI)	RR (95% CI)
Initial mean level difference	1.168 (1.095, 1.246)*	1.213 (1.107, 1.329)*	1.186 (1.089, 1.292)*	1.071 (0.837, 1.371)
Pre-trend Västerbotten	0.949 (0.938, 0.959)*	0.954 (0.948, 0.960)*	0.925 (0.909, 0.940)*	0.954 (0.913, 0.996)*
Pre-trend Norrland	0.969 (0.963, 0.976)*	0.976 (0.963, 0.990)*	0.950 (0.943, 0.957)*	0.965 (0.947, 0.982)*
Pre-trend difference	0.979 (0.966, 0.992)*	0.978 (0.963, 0.992)*	0.973 (0.956, 0.991)*	0.989 (0.943, 1.036)
Level difference	1.024 (0.944, 1.110)	0.981 (0.906, 1.061)	0.978 (0.856, 1.117)	1.004 (0.811, 1.243)
Post-trend Västerbotten	0.976 (0.973, 0.979)*	0.969 (0.964, 0.974)*	0.952 (0.945, 0.959)*	0.942 (0.930, 0.953)*
Post-trend Norrland	0.982 (0.977, 0.987)*	0.979 (0.976, 0.982)*	0.955 (0.948, 0.962)*	0.952 (0.947, 0.957)*
Post-trend difference	0.994 (0.989, 0.999)*	0.990 (0.984, 0.996)*	0.997 (0.987, 1.007)	0.989 (0.976, 1.002)
Pre-Post trend difference	1.016 (1.001, 1.030)*	1.013 (0.996, 1.029)	1.024 (1.004, 1.046)*	1.000 (0.953, 1.050)

*significant at *p* < 0.05

A similar pattern was observed among women with significant differences in the pre-intervention period in the initial mean level, and with differences of the pre-intervention trends slopes in Västerbotten being numerically larger (2.2%) than in the post-intervention periods (1.0%). In contrast to men the corresponding pre-post-trend difference was not significant (RR = 1.013; 95% CI = 0.996, 1.029) (Table 1).

A series of 22 sensitivity analyses (see Additional file 1) applying alternative combinations of control groups and timing of intervention in women and men overall supported the main inferences. However, in contrast to the main analyses, a significant pre-post trend difference was reached in 5/11 analyses in men, and also appeared in 6/11 analyses in women, all in the direction of a disadvantage of Västerbotten following the intervention.

### Intervention impact on IHD mortality

**Fig. 3:**
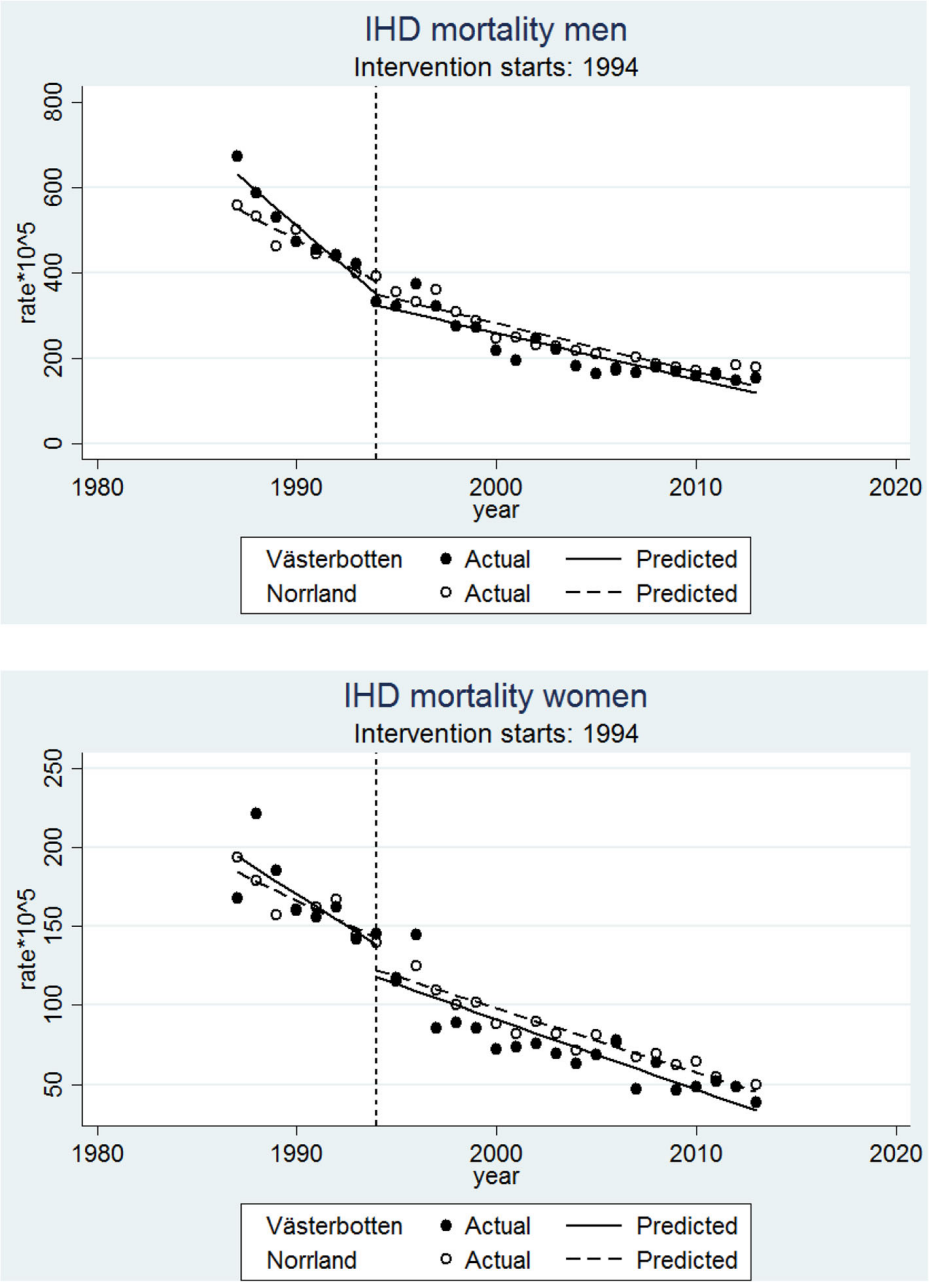
Premature Ischemic Heart Disease (IHD) mortality (per 100,000 population) trends with fitted regression line before (1987–1993) and after (1994–2013) intervention started in the intervention county (Västerbotten) and control counties (Norrland; including Norrbotten, Västernorrland and Jämtland) in men (**a**) and women (**b**)

Results concerning premature IHD mortality are presented in Table 1 and Fig. 3. In men, significant higher mortality was found in Västerbotten compared to the control counties at the beginning of the study (RR = 1.186; 95% CI = 1.089, 1.292), and with Västerbotten displaying a 2.7% larger reduction in mortality before the intervention (pre-trend difference: RR = 0.973; 95% CI = 0.956, 0.991). However, after the intervention year 1994, the trends were similar in Västerbotten and control counties (post-trend difference: RR = 0.997; 95% CI = 0.987, 1.007). This pattern was summarized in a significant pre-post trend differences (RR = 1.024; 95% CI = 1.004, 1.046), reflecting that Västerbotten displayed a significantly smaller reduction in mortality after the intervention than would have been expected from its pre-intervention trend and the trends in the control counties.

In women, a contrasting pattern was seen, with a slightly but not significantly steeper downward mortality trend in Västerbotten than in the control counties, of an identical magnitude (1.1%) before and after the intervention. This pattern resulted in a non-significant pre-post trend (RR = 1.000; 95% CI = 0.953, 1.050), reflecting that the larger mortality reduction in Västerbotten after the intervention was to the same degree as would have been expected from the pre-intervention trend and the trends of the control counties.

Sensitivity analyses (Additional file 1) confirmed this pattern, with significant pre-post trend differences to the disadvantage of Västerbotten after the intervention in 8/11 analyses in men, and no significant pre-post trend differences in any of the analyses in women.

## Discussion

The present study illustrates impressive reductions in premature IHD morbidity and mortality in the total populations of Västerbotten and neighboring counties in Norrland across 27 years. However, the study could not find any evidence of more favorable morbidity or mortality trends in the total population aged 40–74 years in Västerbotten county following the implementation of the Västerbotten Intervention Program in 1993, above and beyond trends before the full implementation of the intervention and the trends in the neighbouring counties which lacked similar programs.

Great improvements in the major cardiovascular risk factors (cholesterol, hypertension, smoking) have already been observed in the population of Northern Sweden since before the full implementation of the VIP, despite the increasing prevalence of obesity [28–30]. Primary prevention interventions at both national and county levels, focusing on healthier diets, anti-smoking campaigns and promptly access to treatment are probably responsible of such trends [3]. This could explain our findings of substantial reductions in IHD occurring not only in Västerbotten but also ubiquitously in the northern Swedish counties, and in Sweden as a whole.

Our results should be seen in the light of a recent study by Blomstedt et al. [19] which evaluated the impact of the VIP model on all-cause and CVD mortality 1990–2006. This was done by comparing both the eligible individuals (all residents of Västerbotten turning 40, 50 or 60 years each year), and the individuals participating in the health checks of the VIP, to the Swedish population by standardised mortality ratios (SMRs). The authors reported significantly lower CVD mortality in both, the target group (3.2% lower rates in men and 9.6% in women, compared to Sweden) and the participants (28.7% in men and 36.6% in women), and even lower all-cause mortality (7.8% in men and 12.1% in women in the target group; and 32.4 and 35.6% in participating men and women respectively). They therefore concluded that the VIP was able to reduce all-cause and CVD mortality, which is conflicting with the results of the present study and warrants some discussion.

One possible reason for the apparent contradiction is that the two studies to varying degree capture the incidence and intervention impact in different subpopulations. Specifically, the population of Västerbotten can be divided into three groups: i) those exposed to the individual-oriented component of the VIP (i.e. participants in the health checks); ii) those targeted by but not exposed to the individual-oriented component (i.e. eligible non-participants); and iii) those neither targeted by nor exposed to the individual-oriented component (i.e. non-eligible non-participants). These subpopulations thus all have in common that they, at least in theory, are exposed to the population-oriented component of the VIP, but differ with regard to whether they are targeted by and exposed to the individual-oriented component; the health checks. From this point of view, the present study captures the impact in all of these three subpopulations without any possibility to differentiate between them. The Blomstedt study, in contrast, captures the impact in participants, as well as in the target group, which comprises both participants and eligible non-participants according to the intention-to-treat principle. As noted above, the Blomstedt study does report an impressive intervention impact on CVD mortality reduction for participants, but a markedly smaller mortality reduction in the combined target group. This strongly suggests that the eligible non-participants actually had higher CVD mortality than the comparator of Sweden, largely offsetting the reduction among participants when analyzed according to the intention-to-treat principle. If a similar pattern of worse-than-expected mortality trends would be also seen in the third group of non-eligible non-participants (which neither of the two studies are able to support or refute) this could completely offset the effect on mortality reduction seen among participants in the health checks. This explanation does not dispute that participation in the VIP health checks are effective in reducing the CVD mortality, as the Blomstedt study demonstrates, but would explain why such an impact is not reflected in reduced IHD morbidity and mortality at the population level, as the present study shows.

There are several possible reasons, both real-world and methodological and both related and unrelated to the implementation of the VIP per se, that alone or in combination could explain such a divergent development of CVD between participant and non-participants of the VIP health checks. First, it is possible that those participating in the health checks of the VIP represent a more healthy population than the (eligible or non-eligible) non-participants, and that the apparent positive impact of the VIP health checks thus, at least partially, is a reflection of selection bias. As has been emphasized in the literature [31, 32], it is highly important that preventive interventions make use of active case-finding strategies to include disadvantaged groups who may face barriers to healthcare such as long distances to facilities, inconvenient appointment schedules and language/communication issues. To the degree that the VIP participants might represent a population of particularly low risk, such active strategies to include vulnerable groups of high CVD risk could improve both the population impact of the VIP and the validity by which it can be evaluated.

Second, it is possible that the implementation of the VIP has led to a concentration of CVD preventive efforts to the VIP health checks, through for example competition of resources and priorities within the county council. If that would be the case, individuals in Västerbotten not participating in the VIP health checks could benefit from preventive efforts to a lesser degree than individuals in the neighboring counties without similar programs, or in Sweden as a whole. Despite that participation rates in the VIP are relatively high [33], to obtain a positive effect through this individual-oriented strategy at the total population level might require an even higher coverage.

Third, compounding the preceding issue, it is possible that a gradual shift within the VIP, from a combined individual- and community-based strategy to a singular individual-based strategy, would leave the non-participants of the health checks especially unattended with respect to cardiovascular health promotion and risk. Researchers working in VIP have indeed reported that the community based component, managed by the municipalities, was very intense and structured in the first years of the intervention but became less intense and unstructured later [14]. This would be especially detrimental for population health if community-based approaches are more effective than individual strategies, as some studies suggest [8, 34], leaving the potential benefit of the VIP isolated to participants in the health checks.

Additionally, if the population-oriented strategies in Västerbotten are eschewed in place of the individual-oriented strategy to an even greater degree than in neighboring counties and Sweden, that could contribute to even less favorable risk of CVD specifically for the non-participants in the VIP health checks.

Looking beyond the northern Swedish case, evaluations in England and Scotland have also found poor evidence of improved identification of undiagnosed disease cases as a result of health checks, but some evidence of decreased CVD risks [12, 35]. Furthermore, a randomized population based study in the city of Copenhagen, where a systematic screening of the general population for high risk followed by lifestyle counselling was implemented during 5 years, found no effect on ischaemic heart disease, stroke, or mortality at the population level after 10 years [36]. These studies are also in line with a recent Cochrane review concluding that there is evidence from general health checks for a reduction in risk factors but not in morbidity or mortality [13]. Taken together, the present study thus adds to the literature illustrating the challenging, and in certain cases possibly futile task, of attempting to achieve an impact on population health through strategies targeting individuals [34].

### Methodological considerations

The present study contains certain methodological strengths and weaknesses that require to be mentioned. Interrupted time series designs, particularly when controlled as in the present study, are considered as the best available means of assessing an intervention impact [22, 23] since it makes full use of the longitudinal nature of the data and account for pre-intervention trends [37]. The study included eight years of observation before the intervention and a follow up period of 19 years, which can be considered enough to capture decreases in morbidity and mortality [22], although the need of more time to observe certain impact cannot be ruled out. Moreover, the analysis was based on aggregated data and can therefore not be used to make inferences about individual-level outcomes.

The use of a valid control group non-exposed to the intervention is key to any evaluative effort and to approach a counterfactual causal scenario, but often difficult to achieve. In most of the analyses, statistical differences between Västerbotten and the control groups were found in the initial mean baseline suggesting that we cannot rule out differences in the groups´ composition that could affect the assumption of exchangeability. It is furthermore difficult to identify competing interventions that could affect the outcomes. CVD prevention programs implemented nationally by the National Institute of Public Health would be expected to have a similar effect over all counties, keeping the VIP as the only exposure, although this assumption may not hold. Finally, while case ascertainment can be an issue, a quality control of the registers is performed routinely on the submitted data, being considered of good quality and with few missing values [38, 39].

## Conclusion

A general decrease in premature ischemic heart disease morbidity and mortality was found in both the intervention county and the neighboring control counties of Northern Sweden. However, the study did not find any evidence for a more positive development in Västerbotten following the implementation of Västerbotten Intervention Program, compared to neighboring counties without similar programs and trends before the intervention.

The findings illustrate the paradox that large-scale preventive efforts based on individual-oriented strategies, even if potentially effective for participating individuals, not necessarily impact positively on population health. Complex CVD prevention programs, such as VIP, therefore require close monitoring and evaluative efforts to better understand the implications of such interventions. Insofar as the goal of the program is to improve population health, population health outcomes also need to be included among the evaluative parameters, and particular attention may need to be paid to the characteristics, pre-intervention health risks and health care access of non-participants.

## Additional file

Additional file 1: 1. IHD MORBIDITY MEN; 2. IHD MORBIDITY WOMEN; 3. IHD MORTALITY MEN; 4. IHD MORTALITY WOMEN. Sensitivity analyses for IHD morbidity and mortality by sex were carried out using a) 1999 and 2004 instead of 1994 as cut-off time points for assessing the trends in all comparison groups; b) the two northern neighbouring counties as separate controls; and c) using national rates as controls; comprising eleven alternative analyses run per outcome and sex, in total 44 analyses. (DOCX 61 kb)

## Abbreviations

95% CI: 95% confidence intervals
BMI: Body Mass Index
CI: Cumulative incidence
CVD: Cardiovascular disease
ICD: International Classification of Diseases
IHD: Ischemic heart disease
ITS: Interrupted time series
RR: Rate ratios
VIP: Västerbotten Intervention Program
