# Supplementary material for: Do cardiovascular disease prevention programs in northern Sweden impact on population health? An interrupted time series analysis
Source: BMC Public Health. 2019 Feb 15;19:202. doi: 10.1186/s12889-019-6514-x (PMC6377762; doi:10.1186/s12889-019-6514-x)
Supplement: Supplementary file 1 — 1. IHD MORBIDITY MEN; 2. IHD MORBIDITY WOMEN; 3. IHD MORTALITY MEN; 4. IHD MORTALITY WOMEN. Sensitivity analyses for IHD morbidity and mortality by sex were carried out using a) 1999 and 2004 instead of 1994 as cut-off time points for assessing the trends in all comparison groups; b) the two northern neighbouring counties as separate controls; and c) using national rates as controls; comprising eleven alternative analyses run per outcome and sex, in total 44 analyses. (DOCX 61 kb) [file 12889_2019_6514_MOESM1_ESM.docx]

**APPENDIX 1**

**1. IHD MORBIDITY MEN**

**Table 1.1.** Segmented linear regression analysis of IHD morbidity trends in men comparing the county of ***Västerbotten with Norrland*** using different time points: 1999, 2004. Results were obtained by negative binomial regression applied to morbidity rates.

|  | Time point 1999 | Time point 2004 |
| --- | --- | --- |
|  |  |  |
| Initial mean level difference | 1.121 (1.054, 1.193)* | 1.108 (1.035, 1.186)* |
| Pre-trend Västerbotten | .953 (.945, .961)* | .968 (.956, 979)* |
| Pre-trend Norrland | .965 (.959, .969)* | .969 (.965, .973)* |
| Pre-trend difference | .988 (.979, .998)* | .991 (.983, .998)* |
|  |  |  |
| Level difference | 1.025 (.931, 1.128) | 1.023 (.921, 1.135) |
|  |  |  |
| Post-trend Västerbotten | .978 (.973, .983)* | .979 (.973, .985)* |
| Post-trend Norrland | .986 (.979 .992)* | .987 (.976, .999)* |
| Post-trend difference | .993 (.985, 1.001) | .991 (.978, 1.005) |
|  |  |  |
| Pre-Post trend difference | 1.004 (.992, 1.017) | 1.000 (.985, 1.016) |

* p-value <0.05

**Table 1.2.** Segmented linear regression analysis of IHD morbidity trends in men comparing the county of ***Västerbotten with Norrbotten*** using different time points: 1994, 1999, 2004. Results were obtained by negative binomial regression applied to morbidity rates.

|  | Time point 1994 | Time point 1999 | Time point 2004 |
| --- | --- | --- | --- |
|  |  |  |  |
| Initial mean level difference | 1.081 (.987, 1.184) | 1.034 (.960, 1.113) | 1.039 (.961, 1.125) |
| Pre-trend Västerbotten | .949 (.938, .959)* | .953 (.945, .961)* | .968 (.956, .979)* |
| Pre-trend Norrbotten | .963 (.948, .978)* | .956 (.949, .964)* | .964 (.959, .970)* |
| Pre-trend difference | .985 (.967, 1.004) | .997 (.986, 1.008) | .995 (.987, 1.004) |
|  |  |  |  |
| Level difference | 1.067 (.964, 1.180) | .979 (.877, 1.094) | .935 (.824, 1.062) |
|  |  |  |  |
| Post-trend Västerbotten | .976 (.973, .979)* | .978 (.973, .983)* | .979 (.973, .985)* |
| Post-trend Norrbotten | .988 (.983, .993)* | .991 (.986, .997)* | .989 (.978, 1.001) |
| Post-trend difference | .988 (.983, .9941)* | .987 (.979, .995)* | .989 (.976, 1.003) |
|  |  |  |  |
| Pre-Post trend difference | 1.003 (.983, 1.023) | .990 (.977, 1.004) | .994 (.978, 1.010) |

* p-value <0.05

**Table 1.3.** Segmented linear regression analysis of IHD morbidity trends in men comparing the county of ***Västerbotten with Västernorrland*** using different time points: 1994, 1999, 2004. Results were obtained by negative binomial regression applied to morbidity rates.

|  | Time point 1994 | Time point 1999 | Time point 2004 |
| --- | --- | --- | --- |
|  |  |  |  |
| Initial mean level difference | 1.299 (1.196, 1.412)* | 1.249 (1.155, 1.350)* | 1.233 (1.138, 1.335)* |
| Pre-trend Västerbotten | .949 (.938, .959)* | .953 (.945, .961)* | .968 (.956, .980)* |
| Pre-trend Västernorrland | .979 (.969, .989)* | .974 (.968, .981)* | .978 (.973, .983)* |
| Pre-trend difference | .969 (.954, .984)* | .979 (.969, .989)* | .981 (.973, .990)* |
|  |  |  |  |
| Level difference | .989 (.911, 1.074) | 1.009 (.912, 1.116) | 1.093 (.979, 1.219) |
|  |  |  |  |
| Post-trend Västerbotten | .976 (.973, .979)* | .978 (.973, .983)* | .979 (.973, .985)* |
| Post-trend Västernorrland | .980 (.976, .985)* | .980 (.972, .988)* | .983 (.969, .997)* |
| Post-trend difference | .996 (.990, 1.001) | .998 (.989, 1.008) | .996 (.981, 1.011) |
|  |  |  |  |
| Pre-Post trend difference | 1.028 (1.011, 1.045)* | 1.020 (1.006, 1.035)* | 1.015 (.997, 1.033) |

* p-value <0.05

**Table 1.4.** Segmented linear regression analysis of IHD mortality trends in men comparing the county of ***Västerbotten with Sweden*** using different time points: 1994, 1999, 2004. Results were obtained by negative binomial regression applied to morbidity rates.

|  | Time point 1994 | Time point 1999 | Time point 2004 |
| --- | --- | --- | --- |
|  |  |  |  |
| Initial mean level difference | 1.303 (1.228, 1.383)* | 1.270 (1.202, 1.343)* | 1.254 (1.176, 1.337)* |
| Pre-trend Västerbotten | .949 (.938, .959)* | .953 (.945, .961)* | .968 (.956, .980)* |
| Pre-trend Sweden | .965 (.958, .971)* | .964 (.961, .967)* | .968 (.965, .971)* |
| Pre-trend difference | .983 (.971, .996)* | .989 (.980, .997)* | .991 (.984, .999)* |
|  |  |  |  |
| Level difference | .981 (.913, 1.053) | 1.013 (.930, 1.104) | 1.031 (.943, 1.128) |
|  |  |  |  |
| Post-trend Västerbotten | .976 (.973, .979)* | .978 (.973, .983)* | .979 (.973, .985)* |
| Post-trend Sweden | .976 (.974, .978)* | .978 (.975, .980)* | .977 (.975, .980)* |
| Post-trend difference | 1.00 (.997, 1.004) | 1.001 (.995, 1.006) | 1.002 (.995, 1.009) |
|  |  |  |  |
| Pre-Post trend difference | 1.017 (1.003, 1.031)* | 1.012 (1.001, 1.023)* | 1.010 (1.00, 1.020)* |

* p-value <0.05

**2. IHD MORBIDITY WOMEN**

**Table 2.1.** Segmented linear regression analysis of IHD morbidity trends in women comparing the county of ***Västerbotten with Norrland*** using different time points: 1999, 2004. Results were obtained by negative binomial regression applied to morbidity rates.

|  | Time point 1999 | Time point 2004 |
| --- | --- | --- |
|  |  |  |
| Initial mean level difference | 1.209 (1.129, 1.296)* | 1.211 (1.127, 1.301)* |
| Pre-trend Västerbotten | .953 (.949, .957)* | .963 (.952, .974)* |
| Pre-trend Norrland | .974 (.967, .980) | .980 (.974, .987)* |
| Pre-trend difference | .979 (.971, .987)* | .979 (.970, .987)* |
|  |  |  |
| Level difference | 1.019 (.916, 1.133) | 1.170 (1.024, 1.336)* |
|  |  |  |
| Post-trend Västerbotten | .969 (.961, .977)* | .956 (.943, .969)* |
| Post-trend Norrland | .976 (.970, .983)* | .977 (.969, .986)* |
| Post-trend difference | .992 (.982, 1.002) | .978 (.962, .994)* |
|  |  |  |
| Pre-Post trend difference | 1.014 (1.00, 1.273)* | .999 (.981, 1.018) |

* p-value <0.05

**Table 2.2.** Segmented linear regression analysis of IHD morbidity trends in women comparing the county of ***Västerbotten with Norrbotten*** using different time points: 1994, 1999, 2004. Results were obtained by negative binomial regression applied to morbidity rates.

|  | Time point 1994 | Time point 1999 | Time point 2004 |
| --- | --- | --- | --- |
|  |  |  |  |
| Initial mean level difference | 1.129 (.941, 1.355) | 1.121 (.997, 1.260) | 1.140 (1.017, 1.278)* |
| Pre-trend Västerbotten | .954 (.949, .960)* | .953 (.949, .957)* | .963 (.952, .974)* |
| Pre-trend Norrbotten | .968 (.931, 1.007) | .965 (.952, .979)* | .975 (.965, .985)* |
| Pre-trend difference | .985 (.947, 1.025) | .987 (.973, 1.001) | .984 (.972, .996)* |
|  |  |  |  |
| Level difference | 1.022 (.845, 1.235) | 1.026 (.891, 1.181) | 1.132 (.951, 1.346) |
|  |  |  |  |
| Post-trend Västerbotten | .969 (.964, .974)* | .969 (.961, .977)* | .956 (.943, .969)* |
| Post-trend Norrbotten | .984 (.978, .991)* | .987 (.978, .996)* | .990 (.975, 1.005) |
| Post-trend difference | .984 (.976, .993)* | .982 (.970, .994)* | .965 (.946, .985)* |
|  |  |  |  |
| Pre-Post trend difference | .999 (.959, 1.040) | .995 (.976, 1.014) | .981 (.957, 1.005) |

* p-value <0.05

**Table 2.3.** Segmented linear regression analysis of IHD morbidity trends in women comparing the county of ***Västerbotten with Västernorrland*** using different time points: 1994, 1999, 2004. Results were obtained by negative binomial regression applied to morbidity rates.

|  | Time point 1994 | Time point 1999 | Time point 2004 |
| --- | --- | --- | --- |
|  |  |  |  |
| Initial mean level difference | 1.336 (1.235, 1.446)* | 1.347 (1.265, 1.435)* | 1.339 (1.255, 1.428)* |
| Pre-trend Västerbotten | .954 (.949, .960)* | .953 (.949, .957)* | .963 (.952, .974)* |
| Pre-trend Västernorrland | .988 (.970, 1.006) | .988 (.981, .995)* | .993 (.989, .998)* |
| Pre-trend difference | .966 (.947, .985)* | .965 (.956, .973)* | .966 (.959, .973)* |
|  |  |  |  |
| Level difference | .912 (.798, 1.042) | .974 (.859, 1.105) | 1.245 (1.079, 1.437)* |
|  |  |  |  |
| Post-trend Västerbotten | .969 (.964, .974)* | .969 (.961, .977)* | .956 (.943, .969)* |
| Post-trend Västernorrland | .975 (.969, .982)* | .964 (.954, .974)* | .962 (.943, .982)* |
| Post-trend difference | .994 (.986, 1.002) | 1.005 (.992, 1.012) | .994 (.969, 1.018) |
|  |  |  |  |
| Pre-Post trend difference | 1.029 (1.008, 1.051)* | 1.042 (1.025, 1.058)* | 1.029 (1.003, 1.055)* |

* p-value <0.05

**Table 2.4.** Segmented linear regression analysis of IHD morbidity trends in women comparing the county of ***Västerbotten with Sweden*** using different time points: 1994, 1999, 2004. Results were obtained by negative binomial regression applied to morbidity rates.

|  | Time point 1994 | Time point 1999 | Time point 2004 |
| --- | --- | --- | --- |
|  |  |  |  |
| Initial mean level difference | 1.373 (1.314, 1.434)* | 1.358 (1.309, 1.408)* | 1.354 (1.292, 1.419)* |
| Pre-trend Västerbotten | .954 (.949, .960)* | .953 (.949, .957)* | .963 (.952, .974)* |
| Pre-trend Sweden | .975 (.971, .979)* | .969 (.967, .972)* | .975 (.972, .979)* |
| Pre-trend difference | .979 (.972, .986)* | .983 (.978, .988)* | .984 (.977, .991)* |
|  |  |  |  |
| Level difference | 1.008 (.944, 1.077) | 1.022 (.930, 1.123) | 1.128 (1.012, 1.259)* |
|  |  |  |  |
| Post-trend Västerbotten | .969 (.964, .974)* | .969 (.961, .977)* | .956 (.943, .969)* |
| Post-trend Sweden | .977 (.975, .979)* | .974 (.969, .979)* | .972 (.967, .977)* |
| Post-trend difference | .992 (.987, .998)* | .994 (.985, 1.004) | .983 (.969, .998)* |
|  |  |  |  |
| Pre-Post trend difference | 1.014 (1.004, 1.023)* | 1.011 (1.00, 1.022)* | .999 (.983, 1.016) |

* p-value <0.05

**3. IHD MORTALITY MEN**

**Table 3.1.** Segmented linear regression analysis of IHD mortality trends in men comparing the county of ***Västerbotten with Norrland*** using different time points: 1999, 2004. Results were obtained by negative binomial regression applied to mortality rates.

|  | Time point 1999 | Time point 2004 |
| --- | --- | --- |
|  |  |  |
| Initial mean level difference | 1.164 (1.075, 1.261)* | 1.123 (1.044, 1.208)* |
| Pre-trend Västerbotten | .930 (.919, .941)* | .936 (.919, .952)* |
| Pre-trend Norrland | .951 (.944, .957)* | .945 (.942, .949)* |
| Pre-trend difference | .978 (.965, .991)* | .985 (.976, .995)* |
|  |  |  |
| Level difference | 1.052 (.885, 1.249) | 1.012 (.872, 1.175) |
|  |  |  |
| Post-trend Västerbotten | .964 (.953, .975)* | .984 (.976, .991)* |
| Post-trend Norrland | .966 (.958, .974)* | .979 (.966, .993)* |
| Post-trend difference | .998 (.984, 1.012) | 1.001 (.989, 1.021) |
|  |  |  |
| Pre-Post trend difference | 1.020 (1.001, 1.041)* | 1.020 (1.001, 1.038)* |

* p-value <0.05

**Table 3.2.** Segmented linear regression analysis of IHD mortality trends in men comparing the county of ***Västerbotten with Norrbotten*** using different time points: 1994, 1999, 2004. Results were obtained by negative binomial regression applied to mortality rates.

|  | Time point 1994 | Time point 1999 | Time point 2004 |
| --- | --- | --- | --- |
|  |  |  |  |
| Initial mean level difference | 1.206 (1.077, 1.349)* | 1.143 (1.048, 1.247)* | 1.159 (1.070, 1.255)* |
| Pre-trend Västerbotten | .925 (.909, .940)* | .930 (.919, .941)* | .936 (.919, .952)* |
| Pre-trend Norrbotten | .956 (.939, .975)* | .946 (.936, .957)* | .951 (.945, .957)* |
| Pre-trend difference | .967 (.943, .991)* | .98 (.97, .99)* | .979 (.969, .990)* |
|  |  |  |  |
| Level difference | 1.047 (.902, 1.215) | .913 (.750, 1.111) | 1.023 (.828, 1.264) |
|  |  |  |  |
| Post-trend Västerbotten | .952 (.945, .959)* | .964 (.953, .975)* | .984 (.976, .991)* |
| Post-trend Norrbotten | .964 (.957, .970)* | .966 (.955, .976)* | .984 (.961, 1.009) |
| Post-trend difference | .988 (.978, .998)* | .998 (.983, 1.014) | .999 (.974, 1.025) |
|  |  |  |  |
| Pre-Post trend difference | 1.022 (.995, 1.050) | 1.016 (.993, 1.039) | 1.020 (.992, 1.049) |

* p-value <0.05

**Table 3.3.** Segmented linear regression analysis of IHD mortality trends in men comparing the county of ***Västerbotten with Västernorrland*** using different time points: 1994, 1999, 2004. Results were obtained by negative binomial regression applied to mortality rates.

|  | Time point 1994 | Time point 1999 | Time point 2004 |
| --- | --- | --- | --- |
|  |  |  |  |
| Initial mean level difference | 1.293 (1.130, 1.479)* | 1.263 (1.128, 1.416)* | 1.185 (1.067, 1.317)* |
| Pre-trend Västerbotten | .925 (.909, .940)* | .930 (.919, .941)* | .936 (.919, .952)* |
| Pre-trend Västernorrland | .959 (.934, .983)* | .958 (.948, .967)* | .947 (.939, .954)* |
| Pre-trend difference | .965 (.936, .994)* | .971 (.956, .986)* | .984 (.973, .995)* |
|  |  |  |  |
| Level difference | .975 (.814, 1.169) | 1.133 (.956, 1.343) | 1.022 (.865, 1.201) |
|  |  |  |  |
| Post-trend Västerbotten | .952 (.945, .959)* | .964 (.953, .975)* | .984 (.976, .991)* |
| Post-trend Västernorrland | .952 (.945, .959)* | .966 (.958, .974)* | .975 (.960, .992)* |
| Post-trend difference | 1.001 (.990, 1.011) | .998 (.984, 1.012) | 1.008 (.990, 1.027) |
|  |  |  |  |
| Pre-Post trend difference | 1.037 (1.004, 1.071)* | 1.028 (1.007, 1.049)* | 1.025 (1.003, 1.047)* |

* p-value <0.05

**Table 3.4.** Segmented linear regression analysis of IHD mortality trends in men comparing the county of ***Västerbotten with Sweden*** using different time points: 1994, 1999, 2004. Results were obtained by negative binomial regression applied to mortality rates.

|  | Time point 1994 | Time point 1999 | Time point 2004 |
| --- | --- | --- | --- |
|  |  |  |  |
| Initial mean level difference | 1.358 (1.254, 1.472)* | 1.326 (1.235, 1.424)* | 1.307 (1.223, 1.396)* |
| Pre-trend Västerbotten | .925 (.909, .940)* | .930 (.919, .941)* | .936 (.914, .952)* |
| Pre-trend Sweden | .945 (.940, .951)* | .945 (.925, .992)* | .944 (.943, .946)* |
| Pre-trend difference | .978 (.962, .995)* | .983 (.972, .996)* | .986 (.978, .995)* |
|  |  |  |  |
| Level difference | .946 (.840, 1.065) | .957 (.820, 1.118) | .978 (.867, 1.103) |
|  |  |  |  |
| Post-trend Västerbotten | .953 (.945, .956)* | .964 (.953, .975)* | .984 (.976, .991)* |
| Post-trend Sweden | .950 (.947, .952)* | .954 (.951, .957)* | .960 (.956, .963)* |
| Post-trend difference | 1.003 (.995, 1.011) | 1.011 (.999, 1.023) | 1.025 (1.017, 1.034)* |
|  |  |  |  |
| Pre-Post trend difference | 1.025 (1.006, 1.045)* | 1.028 (1.010, 1.045)* | 1.039 (1.027, 1.052)* |

* p-value <0.05

**4. IHD MORTALITY WOMEN**

**Table 4.1.** Segmented linear regression analysis of IHD mortality trends in women comparing the county of ***Västerbotten with Norrland*** using different time points: 1999, 2004. Results were obtained by negative binomial regression applied to mortality rates.

|  | Time point 1999 | Time point 2004 |
| --- | --- | --- |
|  |  |  |
| Initial mean level difference | 1.073 (.890, 1.293) | 1.097 (.946, 1.272) |
| Pre-trend Västerbotten | .939 (914, .965)* | .930 (.907, .954)* |
| Pre-trend Norrland | .948 (.941, .956)* | .946 (.942, .951)* |
| Pre-trend difference | .990 (.963, 1.018) | .985 (.971, .999)* |
|  |  |  |
| Level difference | .904 (.718, 1.137) | 1.041 (.827, 1.311) |
|  |  |  |
| Post-trend Västerbotten | .956 (.947, .965)* | .945 (.924, .967)* |
| Post-trend Norrland | .955 (.948, .963)* | .949 (.933, .966)* |
| Post-trend difference | 1.000 (.988, 1.012) | .996 (.968, 1.025) |
|  |  |  |
| Pre-Post trend difference | 1.010 (.980, 1.042) | 1.011 (.979, 1.044) |

* p-value <0.05

**Table 4.2.** Segmented linear regression analysis of IHD mortality trends in women comparing the county of ***Västerbotten with Norrbotten*** using different time points: 1994, 1999, 2004. Results were obtained by negative binomial regression applied to mortality rates.

|  | Time point 1994 | Time point 1999 | Time point 2004 |
| --- | --- | --- | --- |
|  |  |  |  |
| Initial mean level difference | 1.171 (.829, 1.655) | 1.120 (.887, 1.413) | 1.146 (.949, 1.382) |
| Pre-trend Västerbotten | .954 (.913, .996)* | .939 (.914, .965)* | .930 (.907, .954)* |
| Pre-trend Norrbotten | .977 (.930, 1.026) | .945 (.926, .965)* | .943 (.930, 1.320) |
| Pre-trend difference | .976 (.915, 1.042) | .993 (.960, 1.028) | .988 (.969, 1.008) |
|  |  |  |  |
| Level difference | 1.158 (.896, 1.498) | .945 (.705, 1.265) | .998 (.763, 1.306) |
|  |  |  |  |
| Post-trend Västerbotten | .942 (.930, .953)* | .956 (.947, .965)* | .945 (.924, .967)* |
| Post-trend Norrbotten | .959 (.952, .966)* | .966 (.951, .981)* | .957 (.938, .976)* |
| Post-trend difference | .982 (.968, .996)* | .990 (.972, 1.008) | .988 (.959, 1.019) |
|  |  |  |  |
| Pre-Post trend difference | 1.006 (.941, 1.075) | .996 (.959, 1.036) | 1.000 (.964, 1.037) |

* p-value <0.05

**Table 4.3.** Segmented linear regression analysis of IHD mortality trends in women comparing the county of ***Västerbotten with Västernorrland*** using different time points: 1994, 1999, 2004. Results were obtained by negative binomial regression applied to mortality rates.

|  | Time point 1994 | Time point 1999 | Time point 2004 |
| --- | --- | --- | --- |
|  |  |  |  |
| Initial mean level difference | 1.031 (.777, 1.368) | 1.074 (.871, 1.324) | 1.112 (.955, 1.296) |
| Pre-trend Västerbotten | .954 (.913, .996) | .939 (.914, .965)* | .930 (.907, .954)* |
| Pre-trend Västernorrland | .960 (.928, .995)* | .956 (.941, .972)* | .954 (.948, .961)* |
| Pre-trend difference | .993 (.939, 1.049) | .982 (.951, 1.013)* | .975 (.963, .988)* |
|  |  |  |  |
| Level difference | .892 (.684, 1.165) | .873 (.670, 1.138) | 1.153 (.818, 1.623) |
|  |  |  |  |
| Post-trend Västerbotten | .942 (.930, .953)* | .956 (.947, .965)* | .945 (.923, .967)* |
| Post-trend Västernorrland | .949 (.938, .960)* | .948 (.937, .959)* | .947 (.904, .992)* |
| Post-trend difference | .992 (.976, 1.009) | 1.008 (.993, 1.023) | .997 (.947, 1.051) |
|  |  |  |  |
| Pre-Post trend difference | .999 (.943, 1.059) | 1.027 (.991, 1.063) | 1.023 (.969, 1.080) |

* p-value <0.05

**Table 4.4.** Segmented linear regression analysis of IHD mortality trends in women comparing the county of ***Västerbotten with Sweden*** using different time points: 1994, 1999, 2004. Results were obtained by negative binomial regression applied to mortality rates.

|  | Time point 1994 | Time point 1999 | Time point 2004 |
| --- | --- | --- | --- |
|  |  |  |  |
| Initial mean level difference | 1.230 (.967, 1.563) | 1.250 (1.039, 1.503)* | 1.285 (1.114, 1.483)* |
| Pre-trend Västerbotten | .954 (.913, .996)* | .939 (.914, .965)* | .930 (.907, 954)* |
| Pre-trend Sweden | .961 (.951, .972)* | .949 (.943, .955)* | .949 (.946, .952)* |
| Pre-trend difference | .992 (.949, 1.037) | .989 (.962, 1.017) | .983 (.969, .996)* |
|  |  |  |  |
| Level difference | .959 (.789, 1.165) | .893 (.717, 1.111) | 1.161 (.946, 1.426) |
|  |  |  |  |
| Post-trend Västerbotten | .942 (.930, .953)* | .956 (.947, .965)* | .945 (.924, .967)* |
| Post-trend Sweden | .948 (.946, .949)* | .949 (.946, .951)* | .953 (.949, .957)* |
| Post-trend difference | .994 (.981, 1.006) | 1.007 (.998, 1.017) | .992 (.969, 1.015) |
|  |  |  |  |
| Pre-Post trend difference | 1.002 (.956, 1.045) | 1.019 (.989, 1.049) | 1.009 (.982, 1.037) |

* p-value <0.05
